# Impact of thromboprophylaxis on hospital acquired thrombosis following discharge in patients admitted with COVID‐19: Multicentre observational study in the UK

**DOI:** 10.1111/bjh.18874

**Published:** 2023-05-18

**Authors:** Deepa J. Arachchillage, Indika Rajakaruna, Zain Odho, Mike Makris, Mike Laffan, CA‐COVID19 Investigators

**Affiliations:** ^1^ Centre for Haematology Department of Immunology and Inflammation, Imperial College London London UK; ^2^ Department of Haematology Imperial College Healthcare NHS Trust London UK; ^3^ Department of Computer Science University of East London, University Way London UK; ^4^ Department of Biochemistry Royal Brompton Hospital London UK; ^5^ Department of Infection, Immunity and Cardiovascular Disease University of Sheffield Sheffield UK

**Keywords:** anticoagulants, COVID‐19, D‐dimer, hospital acquired thrombosis, Thromboprophylaxis, propensity matching

## Abstract

Post‐discharge thromboprophylaxis in patients admitted with COVID‐19 remains controversial. We aimed to determine the impact of thromboprophylaxis on hospital acquired thrombosis (HAT) in patients (≥18 years) discharged following admission for COVID‐19 in an observational study across 26 NHS Trusts in the UK (01.04.2020–31.12.2021). Overall, 8895 patients were included to the study: 971 patients were discharged with thromboprophylaxis and propensity score matched (PSM) with a desired ratio of 1:1, from patients discharged without thromboprophylaxis. Patients with heparin induced thrombocytopenia, major bleeding during admission and pregnant women were excluded. As expected from 1:1 PSM, no difference was observed in parameters between the two groups, including duration of hospital stay, except the thromboprophylaxis group had a significantly higher proportion who had received therapeutic dose anticoagulation during admission. There were no differences in the laboratory parameters especially D‐dimers between the two groups at admission or discharge. Median duration of thromboprophylaxis following discharge from hospital was 4 weeks (1–8 weeks). No difference was found in HAT in patients discharged with TP versus no TP (1.3% vs. 0.92%, *p* = 0.52). Increasing age and smoking significantly increased the risk of HAT. Many patients in both cohorts had raised D‐dimer at discharge but D‐dimer was not associated with increased risk of HAT.

## Graphical abstract

In this multicentre observational study in the UK assessing the role of thromboprophylaxis on thrombosis following discharge in patients admitted with COVID‐19 showed low rates of events and with no difference in patients receive thromboprophylaxis vs propensity matched cohort of patients with no thromboprophylaxis.

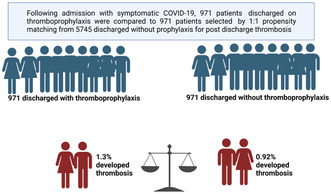

## INTRODUCTION

Coronavirus disease 2019 (COVID‐19), caused by severe acute respiratory syndrome coronavirus 2 (SARS‐CoV‐2), was declared a pandemic by the World Health Organization on March 11, 2020. COVID‐19 progressed through several waves, each with distinct transmission and virulence characteristics. Severe COVID‐19 is associated with an uncontrolled inflammatory response leading to an excess rate of thrombosis including pulmonary thrombosis.

Thromboprophylaxis is given to all in‐patients without contraindications as early evidence from China demonstrated the efficacy of low molecular weight heparin (LMWH) in improving survival.^1^, ^2^ However, it had become standard practice in the UK to perform risk assessment for venous thrombosis (VTE) in all patients admitted to hospital and offer thromboprophylaxis irrespective of COVID‐19 even before the start of the pandemic. The dose of LMWH used in patients admitted with COVID‐19 varied widely from standard dose to intermediate or therapeutic dose depending on the severity of the disease, including in the setting of the multiplatform REMAP‐CAP study.^3^, ^4^ The thrombotic rate in patients admitted to a ward setting was around 6%–14% and this rate was much higher in patients treated in intensive care with reported rates of 22%–34%.^5^, ^6^

Although thromboprophylaxis with LMWH or UFH became the standard practice during the hospital stay, the role of thromboprophylaxis to prevent post discharge hospital acquired thrombosis (HAT), which is defined as VTE up to 90 days following the hospital discharge, was not clear. In general, ~80% of all HATs occur within 6 weeks from hospital discharge in acutely ill medial patients especially in those who required ITU treatment, based on studies prior to COVID‐19.^7^, ^8^ As COVID‐19 is a highly prothrombotic disease, patients with COVID‐19 may continue to have an increased risk of thrombosis following discharge especially if in a high‐risk group (i.e., high D dimer, reduced mobility, prolonged hospital stay including ITU treatment etc).^6^, ^9^ Due to uncertainty over benefit, practice varied widely over the course of the pandemic from no post‐discharge thromboprophylaxis to 7 days–45 days^10^ of thromboprophylaxis with LWMH or direct acting oral anticoagulant.^11^ ISTH guidelines suggested DOAC prophylactic dose to be considered in selected patients.^12^ In a systematic review of National and International Clinical Guidance Reports on of Thromboprophylaxis in Patients with COVID‐19 which included 33 guidance documents (20 published by national and 13 by international societies), extended pharmacological thromboprophylaxis was recommended for patients with high VTE risk after hospital discharge in 63% of documents.^11^

The study by Roberts et al, from King's College London assessed the post discharge VTE rate in patients admitted with COVID‐19 compared to patients without COVID‐19 discharged prior to the COVID‐19 pandemic.^13^ Both groups received thromboprophylaxis only during the hospital stay. Of the 1877 hospital discharges associated with COVID‐19, 9 episodes of VTE were diagnosed within 42 days (4.8 per 1000 discharges) compared to 56 episodes of HA‐VTE within 42 days in 18 159 discharges without COVID‐19 (3.1 per 1000 discharges). The study concluded that COVID‐19 hospitalization does not increase the risk of post discharge VTE compared with hospitalization with other acute medical illness.

Except the above study by Roberts et al, there are no large studies assessing the role of post‐discharge thromboprophylaxis in patients with COVID‐19 admitted to hospitals in the UK. Therefore, to complement the study of Roberts et al we aimed to determine the impact of thromboprophylaxis on hospital acquired thrombosis (HAT) in patients discharged following hospital admission with COVID‐19 in the UK.

## METHODS

This was a multicentre observational study across 26 NHS (national health service) Trusts in the UK. Data were collected both retrospectively and prospectively using a central Redcap database by clinicians directly involving the management of patients (Coagulopathy in COVID19—A Multi‐Centre Observational Study in UK https://www.clinicaltrials.gov/ct2/show/NCT04405232). Adult patients (≥18 years) admitted with symptomatic COVID‐19 between 1 April 2020 and 31 December 2021 were included. All patients had SARS‐CoV‐2 infection confirmed by RT‐PCR positive nasal swabs or nasopharyngeal or lower respiratory tract aspirates. The study was approved by the Human Research Authority (HRA), Health and Care Research Wales and the local Caldicott Guardian in Scotland (20/HRA/1785). Data were collected from patient clinical records by the treating medical team with no breach of privacy or anonymity by allocating a unique study number with no direct patient‐identifiable data; therefore, consent was waived by the HRA. Details on data collection are summarized in Data S1 page 1. At the discharge from hospital, laboratory parameters and use of thromboprophylaxis (type of thromboprophylaxis, dose and the duration) were collected. Post discharge VTE up to 90 days from the hospital discharge and the readmission due to major bleeding events were documented.

### Thromboprophylaxis during the hospital admission

It is standard practice in the UK to give at least prophylactic dose LMWH to all patients admitted to hospital with COVID‐19 unless contraindicated for example by the presence of a bleeding disorder, major bleeding or a platelet count <30 × 10^9^/L, or to continue therapeutic anticoagulation if already on treatment dose anticoagulation.

### Thromboprophylaxis following the hospital discharge

There was no standard approach to thromboprophylaxis across the study centres. The decision to discharge with thromboprophylaxis was based on local guidelines and this varied depending on the NHS trust and the timing of the pandemic.

#### Statistical analysis

Standard descriptive parameters were calculated for categorical and quantitative variables and presented as frequencies with percentages, or medians with a range. Two cohorts of patients (1:1 propensity score‐matched) for patients discharged with thromboprophylaxis and patients discharged without thromboprophylaxis were compared using either the two‐tailed, *t*‐test (numerical parameters), chi‐squared test or chi‐squared trend test (categorial data).

Logistic regression analyses were performed to assess the association between independent variables and development of HAT (yes or no) in the whole study group (both cohorts discharged with thromboprophylaxis and the cohort without thromboprophylaxis together) and the cohort discharged without thromboprophylaxis. Following univariate analyses to identify significant factors associated with development of HAT, multivariate analyses were performed. However, in the final logistic regression models, the following variables were included regardless of their univariate *p‐*values in the univariate analyses: age, body mass index, IMPROVE‐DD VTE score, use of thromboprophylaxis, and D‐dimer >4 to 6 times the ULN (upper limit of normal) or >6 times the ULN, history of VTE prior to admission, coronary artery disease, chronic renal disease, lung disease, ethnicity, mechanical ventilation and ICU admission. Variables with univariate *p* values <0.05 were also included in the final model. Results are reported as adjusted odds ratios (ORs) with 95% confidence intervals (CIs). Details on data management are provided in Data S1 page 1.

## RESULTS

**FIGURE 1:**
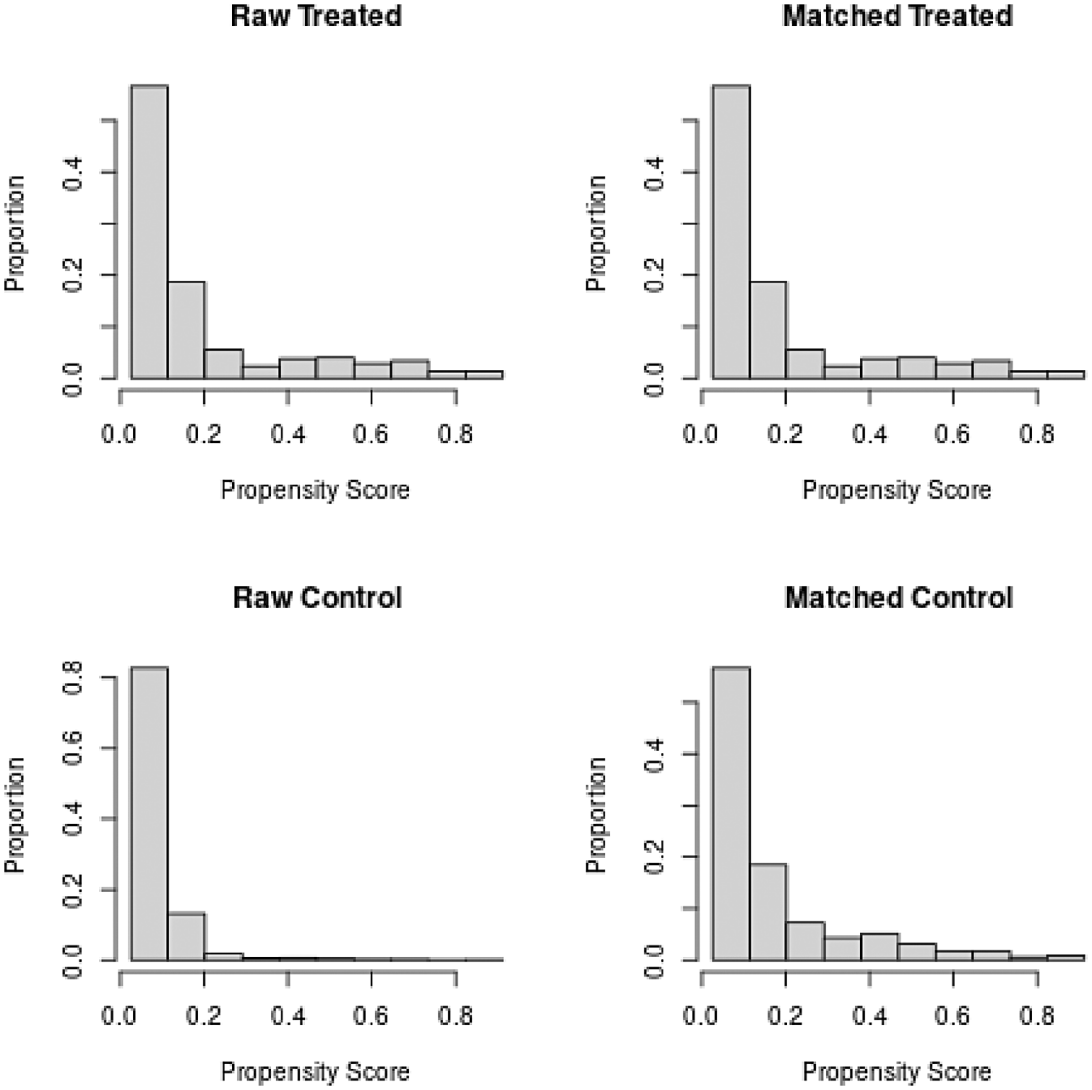
1:1 propensity matching for the patients discharged with thromboprophylaxis versus no thromboprophylaxis.

**FIGURE 2:**
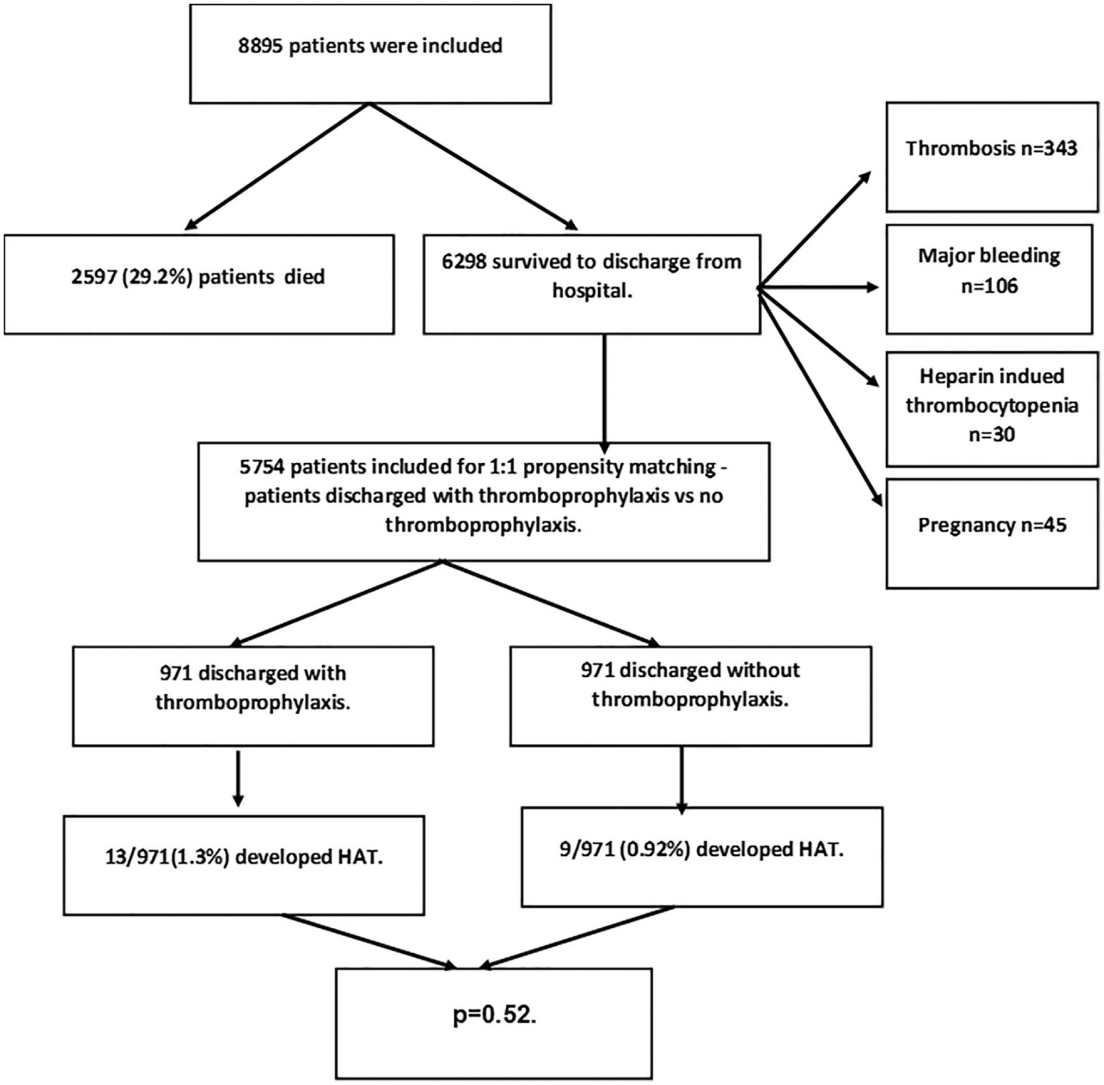
Study population and outcomes.

Overall, 8895 patients were included to the study. Of these 971 patients were discharged with thromboprophylaxis and propensity score matching (PSM) was performed using the nearest‐neighbours method, with a desired ratio of 1:1, from patients discharged without thromboprophylaxis (Figure 1). Patients who had thrombosis, heparin induced thrombocytopenia or major bleeding (defined according to International Society on Thrombosis and Haemostasis (ISTH) classification^14^) during admission and pregnant women were excluded (Figure 2). Comparison of demographics and comorbidities between the propensity matched cohort of patients discharged with thromboprophylaxis versus the no thromboprophylaxis group is presented in Table 1.

**TABLE 1:** Demographics and baseline clinical characteristics of the patients discharged with no thromboprophylaxis versus patients discharged with thromboprophylaxis at admission and discharge.

Demographics	No thromboprophylaxis at discharge	Thromboprophylaxis at discharge	*p* value
Gender			
Men	536	547	0.64
Women	435	424	
Age (years)			
0–29	22	20	0.98
30–49	116	118	
50–69	284	283	
70–89	470	470	
90+	79	80	
BMI (Kg/m^2^)			
0–18.5	18	18	0.39
18.6–24.9	248	238	
25–29.9	363	349	
30–39.9	299	326	
40+	43	40	
Ethnicity			
White	751	748	0.49
Mixed multiple ethnic	8	2	
Asian/Asian British	46	40	
Black African/Caribbean	25	24	
Other ethnic group	25	33	
Unknown	116	124	
Patients on antiplatelet treatment prior to admission			
No	814	814	1
Yes	157	157	
Patients was on anticoagulation prior to admission			
No	841	849	0.58
Yes	130	122	
History of VTE prior to admission			
No	931	921	0.2755
Yes	40	50	
History of autoimmune disease			
No	898	896	0.8649
Yes	73	75	
History of malignancy in the past 6 months prior to admission			
No	863	859	0.7775
Yes	108	112	
Hypertension			
No	589	579	0.6092
Yes	382	392	
Hypercholesterolemia			
No	840	851	0.4596
Yes	131	120	
Ischaemic heart disease			
No	784	775	0.6054
Yes	187	196	
Diabetes mellitus			
No	699	696	0.877
Yes	272	275	
History of smoking			
None	373	371	0.8907
Current smoker	93	100	
Ex‐smoker	212	186	
Unknown	293	314	
History of liver disease			
No	941	931	0.2115
Yes	30	40	
History of lung disease			
No	750	737	0.4778
Yes	221	234	

As expected, there were no differences in the age, gender, demographics or comorbidities between the propensity matched cohorts. Comparison of the laboratory parameters between the two cohorts is summarized in Table 2. Although there were no differences in the inflammatory markers including C‐reactive protein and the D‐dimer levels between the two cohorts, a large majority of patients in both cohorts had raised C‐reactive protein and D‐dimer levels at discharge (83.5% [811/971] vs. 87.3% [848/971] *p* = 0.79 for D‐dimers and 75.2% [730/971] vs. 77.1% [749/971] *p* = 0.61 for C‐reactive protein for patients discharged with or without thromboprophylaxis). No differences in the laboratory parameters were observed between the two cohorts of patients. Table 3 summarizes the interventions and the events during the hospital stay comparing the patients discharged with thromboprophylaxis versus patients discharged without thromboprophylaxis. Except for the higher proportion of patients discharged with thromboprophylaxis who received treatment dose anticoagulation (with no history of thrombosis) (355/971, 36.5%) compared to patients discharged without thromboprophylaxis (182/971, 18.7%), *p* < 0.0001, there were no differences in the two cohorts including the duration of the duration of the hospital stay (Table 3).

**TABLE 2:** Laboratory blood results of the patients discharged with no thromboprophylaxis versus patients discharged with thromboprophylaxis at admission and discharge.

Laboratory parameter	At admission			At discharge		
	No thromboprophylaxis at discharge	Thromboprophylaxis at discharge	*p* value	No thromboprophylaxis at discharge	Thromboprophylaxis at discharge	*p* value
Lactate (mmol/L)						
Normal (0.5–2.1)	879	907	0.72	745	744	0.29
Above normal (>2.1)	81	52		115	102	
Haemoglobin (g/L)^a^						
Below normal <130 (<115)	471	449	0.41	554	542	0.84
Normal 130–160 (115–150)	421	430		285	281	
Above normal >160 (>150)	48	67		23	25	
Lactate dehydrogenase (IU/L)						
Below normal (<266)	24	17	0.11	24	17	0.10
Normal (266–500)	523	510		472	454	
Above normal (>500)	422	434		388	396	
Troponin I (ng/L)						
Normal <19.8	270	241	0.40	259	246	0.48
Above normal >19.7	700	730		626	622	
Ferritin (μg/L)						
Below normal (<20)	3	0	0.65	2	0	0.74
Normal (20–186)	24	22		25	25	
Above normal (>186)	943	949		858	843	
Prothrombin time (s)						
Below normal (<10.2)	10	1	0.40	6	3	0.53
Normal (10.2–13.2)	192	170		200	164	
Above normal (>13.2)	747	778		662	681	
APTT (s)						
Below normal (<26.0)	86	61	0.82	84	56	0.15
Normal (26–36)	761	782		690	684	
Above normal (>36.0)	116	124		105	123	
White cell count (10^9^/L)						
Below normal (<4.1)	79	94	0.16	71	73	0.52
Normal (4.1–11.1)	668	670		610	609	
Above normal (>11.1)	217	197		198	180	
Neutrophils (10^9^/L)						
Below normal (<2.1)	37	47	0.35	40	44	0.72
Normal (2.1–6.7)	507	493		489	466	
Above normal (>6.7)	419	423		341	345	
Fibrinogen (g/L)						
Below normal (<1.5)	17	1	0.92	21	4	0.18
Normal (1.5–4.5)	72	114		84	120	
Above normal (>4.5)	874	845		769	735	
Creatinine (μmol/L)						
Below normal (<60)	136	140	0.42	221	200	0.76
Normal (60–120)	594	577		504	492	
Above normal (>120)	223	242		141	162	
CRP (mg/L)						
Normal (0–10)	93	81	0.34	222	241	0.61
Above normal (>10)	873	882		749	730	
Platelets (10^9^/L)						
Below normal (<150)	158	169	0.16	100	110	0.47
Normal (150–400)	728	724		732	737	
Above normal (>400)	79	73		139	124	
D‐dimer (ng/mL)						
Normal (0–500)	51	64	0.66	123	160	0.71
>500–1000	166	150		413	402	
>1000–2000	249	268		289	272	
>2000–4000	274	250		120	112	
>4000	231	239		26	25	

Abbreviation: APTT, activated partial thromboplastin time.

^a^ Values in brackets indicate normal reference range for female.

**TABLE 3:** 1:1 propensity matching and the comparison between patients discharged with thromboprophylaxis versus no thromboprophylaxis: Clinical interventions and events at or during the admission.

Interventions/events at or during admission	No thromboprophylaxis at discharge	Thromboprophylaxis at discharge	*p* value
Thromboprophylaxis on admission			
No	547	517	0.15
Yes	424	454	
Therapeutic anticoagulation during admission			
No	789	616	**<0.0001**
Yes	182	355	
Steroids during admission			
No	771	779	0.54
Yes	200	192	
Blood transfusion during admission			
No	844	835	0.51
Yes	127	136	
Development of renal failure during admission			
No	915	905	0.29
Yes	56	66	
Clinically significant non‐major bleeding during admission^a^			
No	935	931	0.64
Yes	36	40	
Multiorgan failure during admission			
No	931	921	0.25
Yes	40	50	
Secondary infection during admission			
No	759	764	0.75
Yes	212	207	
Mechanical ventilation during admission			
No	822	810	0.36
Yes	149	161	
Requirement for intensive care unit admission			
No	759	753	0.67
Yes	212	218	
Duration of hospital stay			
Median (range)	32 (3–190)	32 (3–187)	0.98

*Note*: *p* values <0.05 are considered as significant and shown in bold.

^a^ Defined as per ISTH criteria for clinically relevant non‐major bleeding.^24^

### Thromboprophylaxis following hospital discharge

The majority of these patients were discharged with a direct acting oral anticoagulant (DOACs) (496/971, 51.1%) as thromboprophylaxis followed closely by low molecular weight heparin (LMWH) (475/971, 48.9%). Rivaroxaban was the most frequently used DOAC (262/496, 52.8%) followed by apixaban (224/496, 45.2%) and rest of the patients were discharged with dabigatran (10/496, 2.0%).

### Thrombotic events following discharge from hospital (HAT)

Of patients discharged with thromboprophylaxis, 13/971 (1.3%) developed HAT compared to nine patients discharged without thromboprophylaxis (0.92%, 9/971), *p* = 0.52. Of the 13 patients who developed HAT following discharge with thromboprophylaxis, five patients (38.5%) developed pulmonary embolism (PE) and the remainder (61.5%) developed deep vein thrombosis (DVT) of the lower limbs. Nine patients developed HAT when discharged without thromboprophylaxis, three patients (33.3%) developed PE and other 6 patients (66.4%) developed DVT. None of the patients in either group presented to hospital with major bleeding events following discharge.

#### Factors associated with HAT

Factors that can be associated with HAT were assessed separately in the whole study group and in the cohort of patients discharged without thromboprophylaxis. In univariate analysis, age, history of no smoking and D‐dimer >6 times ULN were significantly associated with development of HAT in the overall cohort but only age was significantly associated with developing HAT in the group of patients discharged without thromboprophylaxis.

In multivariate analysis increasing age (odds ratio [OR] 1.08 [95% CI 1.01–1.12], *p* = 0.017) and history of no smoking (OR 0.68 [95% CI 0.47–0.95], *p* = 0.031) were the only significant factors linked with increased risk of HAT in the overall cohort whilst age was the only significant factor associated with HAT in patients discharged without thromboprophylaxis (OR = 1.09 [95% CI 1.03–0.1.13], *p* = 0.013). D‐dimer was not associated with the development of HAT in the multivariate analysis in either group. The distribution of D‐dimer was not normal; hence, we assessed the D dimer as a categorical variable. However, analysis was performed as continuous variable as well which did not show a significant association with HAT. Furthermore, IMPROVE‐DD VTE score (OR 1.5 [95% CI 0.89–2.7]) and D‐dimer >4 to 6 times the ULN (upper limit of normal) (OR 1.7 [0.9–2.1]) or >6 times the ULN (OR 1.9 [0.8–2.3]) at dischage were not associated with increased risk of HAT events. Table 4 summarizes the IMPROVE VTE scores between the two groups. An analysis was performed to assess whether including the highest D dimer value during the hospital stay would change the IMPROVE‐DD scores between the groups and the final multivariate model, but it was not altered.

**TABLE 4:** IMPROVE‐VTE score in patients discharged with thromboprophylaxis versus no thromboprophylaxis.

IMPROVE‐VTE score	No thromboprophylaxis at discharge	Thromboprophylaxis at discharge	*p* value
IMPROVE‐VTE score of ≥4	226	223	0.91
IMPROVE‐VTE score of 2–3 with D dimer >500 ng/mL^a^	541	560	0.40

^a^ D dimer level at discharge.

## DISCUSSION

In this large multicentre observational study assessing the role of thromboprophylaxis on the development of thrombosis (HAT) in patients admitted with COVID‐19, it was found that the rate of HAT is low in patients discharged following COVID‐19, and thromboprophylaxis at discharge did not have a significant impact. In the 1:1 propensity matched cohort of patients discharged without thromboprophylaxis, except for the higher proportion of patients discharged with thromboprophylaxis receiving treatment dose anticoagulation during admission (with no history of thrombosis), there were no differences in the two cohorts, including the duration of the duration of hospital stay. This suggests that if the patient received treatment dose thromboprophylaxis during hospital they were more likely to be also given post discharge thromboprophylaxis. This indicates that at some point these patients were assessed as being at higher risk. However, it was not possible to clarify how the clinicians came to that conclusion.

The median duration of thromboprophylaxis was 4 weeks with a range of 1–8 weeks, reflecting the variability of clinical practice across NHS Trusts regarding thromboprophylaxis. DOACs were the most frequently used anticoagulant as thromboprophylaxis closely followed by LMHW and rivaroxaban was the most prescribed DOAC. In multivariate analysis increasing age was significantly linked with increased risk of HAT whilst history of no smoking reduced the risk by 32% in the overall cohort whilst age was the only significant factors associated with HAT in patients discharged without thromboprophylaxis. It is possible that clinicians were able to correctly identify the lower‐risk patients based on overall clinical impression at discharge although the propensity matching includes multiple measures of disease severity. In multivariate analysis D‐dimer on the day of the discharge from hospital was not associated with the development of HAT in either group as a categorical or continuous variable. Furthermore, including the highest D‐dimer value during the hospital stay did not change the IMPROVE‐DD scores between the groups or the results of the final multivariate model.

Hospital acquired VTE is a global health issue and a systematic approach in risk assessment and thromboprophylaxis during hospital admission has significantly reduced HAT and its related morbidity and mortality.^15^ COVID‐19 is associated with significantly higher risk of thrombosis due to multiple mechanisms including hyperinflammation and endothelial activation.^16^, ^17^ Open‐label, adaptive, multiplatform, controlled trials in hospitalized, not critically ill, patients with COVID‐19 demonstrated that therapeutic‐dose anticoagulation with heparin had better survival and reduced use of organ support (cardiovascular or respiratory) compared to standard dose heparin thromboprophylaxis.^4^ Trials on the use of extended thromboprophylaxis in medically ill patients without COVID‐19 at discharge have demonstrated mixed results, either from not achieving their primary end point or having had an increase in major bleeding.^18^ A meta‐analysis of these trials revealed an overall 39% reduction of symptomatic VTE and VTE‐related death but two‐fold increase in major and fatal bleeding with no significant difference in VTE‐related death.^19^ However, the absolute rates of major bleeding are much lower in patients discharged following COVID‐19, such that there may be a favourable number needed to treat (NNT) when compared to the number needed to harm (NNH).

CORE‐19 is a large prospective registry from New York including 4906 consecutive adult, non‐ obstetric hospitalized COVID‐19 patients from first the wave.^20^ Post discharge thromboprophylaxis was prescribed in 13.2% patients. The primary outcome of the study was a composite of adjudicated VTE, arterial thromboembolism (ATE) and all‐cause mortality (ACM). The primary safety outcome was major bleeding. Rates of VTE, ATE and ACM were 1.55%, 1.71% and 4.83%, respectively. Major bleeding occurred in 1.73%. Composite primary outcome rate was 7.13% and was significantly associated with advanced age, prior VTE, intensive care unit (ICU) stay, chronic kidney disease, peripheral arterial disease, carotid occlusive disease, IMPROVE‐DD VTE score ≥4 and coronary artery disease. Postdischarge thromboprophylaxis was associated with reduction in primary outcome by 46% (CI, 0.47%–0.81%). A meta‐analysis of 18 949 patients with COVID‐19 admitted to hospitals showed a pooled incidence of post‐discharge VTE of 1.8% (95% CI: 0.8%–4.1%).^21^ In comparison with the above studies, VTE rates following discharge in this study were lower (1.3% in the patients discharged with thromboprophylaxis versus 0.92% in patients discharged without thromboprophylaxis). Moreover, none of the patient presented with major bleeding in contrast to 1.73% patients with major bleeding in the CORE‐19 registry.^20^ However, the present study did not assess the all‐cause mortality or ATE.

The MICHELLE trial was an open labelled multicentre randomized trial of post‐discharge extended thromboprophylaxis in COVID‐19 inpatients where 320 patients were randomized to receive rivaroxaban 10 mg daily for 35 days versus no anticoagulation (160 patients in each arm).^22^ All patients received standard doses of heparin thromboprophylaxis during hospitalization. This study included a high thrombotic risk population (IMPROVE VTE score of ≥4 or a score of 2–3 with D dimer >500 ng/mL). Only 159 patients in each arm of the study were included in the intention‐to‐treat primary analysis due to loss of follow‐up. Symptomatic or fatal VTE and/or arterial thrombosis occurred in 3% of rivaroxaban arm versus 9% of no anticoagulation (relative risk 0.33, 95% CI 0.12–0.90; *p* = 0.029). No major bleeding occurred in either study group.^22^ In contrast to the present study, in the MICHELLE trial there was a significant reduction in symptomatic VTE in patients who received rivaroxaban at discharge with no fatal VTE^22^ (1/159 [0.63%] vs. 8/159 [5·03%] with 3/8 fatal events). Compared to the MICHELLE trial where rivaroxaban 10 mg was given for 35 days in the thromboprophylaxis group,^22^ rivaroxaban, apixaban, dabigatran or LMWH were all used as thromboprophylaxis in the present study, and duration varied from 7 days to 56 days with a median 28 days. The MICHELLE trial concluded that inpatients with cardiovascular risk factors, advanced age, intensive care unit stay, or an IMPROVE VTE score of 4 or more or a score of 2 or 3 plus elevated D‐dimers (>2X ULN) or an IMPROVE‐DD VTE score of ≥4 are at high thrombotic risk in the post‐discharge period and may benefit from post discharge thromboprophylaxis.^22^ However, except increasing age and smoking status, we failed to identify other significant factors that are associated with increased risk of post discharge VTE. Interestingly, we did not find a link between raised D dimer and risk of post discharge VTE. At discharge 83.5% (811/971) and 87.3% (848/971) patients had raised D‐dimer in the thromboprophylaxis and no thromboprophylaxis group respectively with no difference between the two. The elevated D‐ dimers suggest ongoing inflammatory response post‐discharge in COVID‐19 patients. This is further supported by the raised C reactive protein levels in 75.2% (730/971) versus 77.1% (749/971) in the two groups. Neither study found significant difference in the major bleeding rates in patients receiving thromboprophylaxis versus no thromboprophylaxis.^22^

The median length of stay in hospital for both groups was 32 days which is much longer than average length of 4.5 days in‐hospital stay for non‐COVID medical patients. Studies assessing the VTE risk in medically ill patients demonstrated that most of VTE events occur within 6 weeks of hospitalization.^7^, ^8^ The lower rates of HAT in patients may be attributed to a longer period of in‐hospital prophylaxis. However, in‐hospital rates of thrombosis were higher for COVID‐19 and the raised D‐dimer and CRP suggest that inflammation had not resolved at time of discharge. Similarly, patients with COVID‐19 were kept in longer because they were ill longer. Therefore, their period of risk was longer and so not comparable to less ill medical patients with shorter admissions.

Some of the patients who were on anticoagulation prior to admission with COVID‐19 were not discharged with thromboprophylaxis (Table 1). Reasons for discharging patients off anticoagulation when they were admitted on anticoagulation were not available. However, there was no difference in the number of patients who stopped their anticoagulant at discharge between the two groups and so the results of our study would not be affected by this. Furthermore, none of the patients who stopped anticoagulant developed HAT following discharge.

https://clinicaltrials.gov/ct2/show/NCT04650087 was an adaptive, prospective, randomized platform trial which compares the safety and efficacy of thromboprophylaxis (apixaban 2.5 mg bd) versus no thromboprophylaxis following hospitalization ≥48 h for COVID‐19. This study terminated early following the recruitment of 1217 participants due to lower event rates and decreasing COVID‐19 hospitalizations. The incidence of the primary end point (30‐day composite of death, arterial thromboembolism and venous thromboembolism) was 2.13% (95% CI, 1.14 to 3.62) in the apixaban group and 2.31% (CI, 1.27 to 3.84) in the placebo group. Similar to our study, the symptomatic VTE rates were low in both study arms (5/607, 0.82% in the placebo arm vs. 5/610, 0.82% in apixaban arm) with no difference. The primary safety end point (major bleeding) occurred in 2 (0.4%) and 1 (0.2%) apixaban‐treated and placebo‐treated participants, respectively.^23^ The observed low rate of venous thrombosis and no difference in patients discharged with thromboprophylaxis versus no thromboprophylaxis are in keeping with the current study.

Our study has some important limitations. It is possible that not all VTE events were captured following hospital discharge although in the UK, there is robust system to assess the post discharge VTE from the ongoing quality improvement program incorporating root‐cause analysis of hospital associated VTE. Even if the patient is not admitted to the same hospital with post discharge VTE, in root‐cause analysis process this data is captured from the admission hospital. During the COVID‐19 pandemic this may not have happened as expected due to reduced resources due to redeployment of the staff. Therefore, our data may underestimate the post‐discharge VTE rate. However, this is applicable to both cohorts in the study. It is not possible to exclude the possibility that patients identified as high‐risk for thrombosis were given more intense prophylaxis in‐hospital as well as thromboprophylaxis post‐discharge using some measure not captured by the propensity matching. ISTH criteria for defining the clinically relevant non‐major bleeding may not be appropriate. However, the same criteria were applied to both groups and the number of patients who developed clinically relevant non‐major bleeding in the two groups were comparable and very small (3.7% in the no thromboprophylaxis at discharge vs. 4.1% Thromboprophylaxis at discharge).

Additionally, this study did not have information on different coronavirus variants over the course of the pandemic which may affect the risk of thrombosis.

Despite the above limitations, this is the largest multicentre study to date assessing the role of post discharge thromboprophylaxis in patients with COVID‐19 representing patients discharged from 26 NHS Trusts across the UK. By including a 1:1 propensity matched cohort of patients with COVID‐19 discharged from the same hospitals with the study period, we have matched the comparator group in the best possible scenario outside a randomized control study. Furthermore, we included the use of steroids, blood transfusion, development of secondary infections, renal, respiratory failure and multiorgan failure which are all known risk factors for development of thrombosis in the propensity matching compared to other studies.^13^, ^20^, ^22^

In conclusion, the rate of HAT is low in patients discharged following COVID ‐19, and thromboprophylaxis at discharge did not have a significant impact on this. Although the majority of patients in both cohorts had raised D‐dimer at discharge D‐dimer was not associated with increased risk of HAT.

## AUTHOR CONTRIBUTIONS

Deepa J. Arachchillage conceived the study, acquired the funding, involved in data collection, data verification, data analysis, figures, data interpretation, writing the original draft reviewing and editing the manuscript. Indika Rajakaruna and Zain Odho were involved in data verification, data analysis, figures, data interpretation and reviewing the manuscript. MM contributed to data collection, interpretation of the data and reviewing the manuscript ML interpreted the data, reviewed, and edited the manuscript. All authors reviewed and approved the final version of the manuscript.

## FUNDING INFORMATION

Bayer PLC supported the study by providing the investigator‐initiated funding (P87339) to setup the multicentre database of the study. The funder had no access to data and played no part in analysis or writing. The corresponding author is responsible for the study design, had full access to all the data in the study and had final responsibility for the decision to submit for publication. DJA is funded by MRC UK (MR/V037633/1).

## CONFLICT OF INTEREST STATEMENT

DJA received funding from Bayer PLC to setup the multicentre database of the study as an investigator‐initiated funding and received speaker fees. ML received speaker fees from Pfizer, Leopharma, Bayer PLC and consultancy fees from Pfizer. Other authors have no conflict of interest to declare.

## Supporting information


Data S1.

## Data Availability

The data that support the findings of this study are available on request from the corresponding author, [DJA] upon reasonable request.

## CA‐COVID19 STUDY COLLABORATORS

Aneurin Bevan University Health Board: Amanda Dell, Angela Hall, Anna Roynon, Anne Heron, Cheri Price, Claire Price, Clare Westacott, Debra Barnett, Gail Marshall, Gemma Hodkinson, Georgia Mallison, Grace Okoro, Joshua Gwatkin, Kirstin Davies, Lucy Shipp, Maxine Nash, Rhian Hughes, Rina Mardania and Sarah Lewis Sean Cutler. Aberdeen Royal Infirmary: Caroline Allan. Barts Health NHS Trust: Atiqa Miah, Dide Okaygun, Dan Hart, Faith Dzumbunu, James Leveson, Karen Torre, Louise Taylor, Priyanka Raheja, Sara Mamsa and Tasnima Ferdousi. Buckinghamshire Healthcare NHS Trust: Angharad Everden, Alice Ngumo, Doaa Ahmed, Efstathia Venizelou, James Herdman, Janice Carpenter, Konrad Bartkiewicz and Rebecca Cash Renu Riat. Cardiff and Vale University Health Board: Abigail Downing, Ana Guerrero, Astrid Etherington, Chapa Gamage, Dilupa Gunasekara, Lee Morris, Raza Alikhan, Rebecca Cloudsdale, Samya Obaji, Stuart Cunningham and Sylvain Ndjombo. County Durham and Darlington NHS Foundation Trust: Amanda Cowton, Ami Wilkinson, Andrea Kay, Anne Sebakungu, Anne Thomson, Clare Brady, Dawn Egginton, Ellen Brown, Enid Wright, Gill Rogers, Hannah Plaschkes, Jacqui Jennings, Julie O'Brien, Julie Temple, Kathryn Potts, Kimberly Stamp, Kelly Postlethwaite, Louise Duncan, Margaret Randall, Mark Birt, Melanie Kent, Philip Mounter, Shelly Wood, Nicola Hewitson, Noreen Kingston, Susan Wadd, Sarah McAuliffe, Stefanie Hobson, Susan Riley, Suzanne Naylor and Vicki Atkinson. Cwm Taf Morgannwg University Health Board: Alysha Hancock, Bethan Deacon, Carla Pothecary, Caroline Hamilton, Ceri Lynch, Cerys Evenden, Deborah Jones, Ellie Davies, Felicity Page, Gareth Kennard‐Holden, Gavin John, Joanne Pugh, Joelle Pike, Justyna Mikusek, Kevin Agravante, Kia Hancock, Lauren Geen, Meryl Rees and Natalie Stroud. Gateshead Health NHS Foundation Trust: Amanda Grahamslaw, Amanda Sanderson, Beverley McClelland, Caitlin Barry, Elaine Siddle, Lorraine Pearce, Lucy Blackwell, Maria Bokhari, Maureen Armstrong, Wendy Stoker and Wendy McCormick. Guy's and St Thomas' NHS Foundation Trust: Caterina Vlachou, Ben Garfield, Mihaela Gaspar, Maurizio Passariello, Paolo Bianchi and Stephane Ledot. Hampshire Hospitals NHS Foundation Trust: Aileen Madlin, Kerrianne Everard, Khushboo Panwar, Natasha Beacher, Niamh Cole, Sarah Mangles, Tamara Everington and Udaya Reddy. Imperial College Healthcare NHS Trust: Alka Shah, Anna Weatherill, Anthi Maropoulou, Bhagya Herath, Billy Hopkins, Camelia Vladescu, Caroline Ward, Christina Crossette‐Thambiah Donna Copeland, Emily Pickford, Gaurika Kapoor, Isabella Lo, John Kilner, Keith Boland, Melanie Almonte, Neil Simpson, Niamh Bohnacker, Omolade Awomolo, Roochi Trikha, Samina Hussain, Serah Duro, Sophie Kathirgamanathan, Yasmine Needham, Yee Hui, Zainab Alashe; King's College Hospital NHS Foundation Trust: Adrienne Abioye, Aileen Miranda, Christina Obiorah, Cynthia Dzienyo, Hasina Mangal, Hernan Zorraquino, Lara N Roberts, Mariusz Racz, Maclaine Hipolito Johnson, Rachel Ryan, Tamara Swales, Tatiana Taran, Zoe Renshaw; Newcastle Hospitals NHS Foundation Trust: Alexander Langridge, Benjamin Evans, Callum Weller, Claire Judd, Douglas Jerry, Euan Haynes, Fatima Jamil, Ian McVittie, John Hanley, Julie Parker, Kayleigh Smith, Keir Pickard, Laura Kennedy, Meghan Acres, Mikaela Wiltshire, Nitha Ramachandran, Paul McAlinden, Paula Glancy, Smeera Nair, Tarek Almugassabi, Thomas Jarvis; NHS Grampian: Amanda Coutts, Andrew Laurie, Deborah Owen, Ian Scott, Jamie Cooper, Leia Kane, Lucy Sim, Mahmoud Abdelrahman and Victoria Poulton. Norfolk and Norwich University Hospitals NHS Foundation Trust: Jessica Griffin, Ria Markwell, Suzanne Docherty; North Cumbria Integrated Care NHS Foundation Trust: Alexander Brown, Barbara Cooper, Beverley Wilkinson, Diane Armstrong, Grace Fryer, Jane Gregory, Katherine Davidson, Melanie Clapham, Nicci Kelsall, Patricia Nicholls, Rachel Hardy, Roderick Oakes, Rosemary Harper, Sara Abdelhamid, Theresa Cooper, Una Poultney and Zoe Saunders. North Tees and Hartlepool NHS Foundation Trust: Alex Ramshaw, Alison Chilvers, Barbara Jean Campbell, Carol Adams, Claire Riley, Deborah Wilson, Helen Wardle, Jill Deane, Jill Skelton, Julie Quigley, Leigh Pollard, Liz Baker, Lynda Poole, Maria Weetman, Michele Clark, Nini Aung, Rachel Taylor, Sarah Rowling, Sarah Purvis and Vicky Collins. Northumbria Healthcare NHS Foundation Trust: Amy Shenfine, Catherine Ashbrook‐Raby, Charlotte Bomken, Claire Walker, Faye Cartner, Helen Campbell, Jane Luke, Jessica Reynolds, Mari Kilner, Laura Winder, Linda Patterson, Lisa Gallagher, Nicola McLarty, Sandra Robinson, Steve Dodds, Toni Hall and Victoria Wright. Oxford University Hospitals NHS Foundation Trust: Agnes Eordogh, Alexandros Rampotas, Anna Maria Sanigorska, Christopher Deane, Kristine Santos, Olivia Lecocq, Rochelle Lay, Simon Fletcher, Susie Shapiro. Royal Free London NHS Foundation Trust: Anna Tarnakina, Aniqa Tasnim, Anja Drebes, Cecilia Garcia, Elsa Aradom, Mariarita Peralta, Michaella Tomlin, Pratima Chowdary, Ramona Georgescu, Suluma Mohamed and Upuli Dissanayake. Royal Liverpool and Broadgreen University Hospitals NHS Trust: Carol Powell, James Doolan, Jessica Kenworthy, Joanne Bell, Lewis Jones, Mikiko Wilkinson, Rebecca Shaw, Ryan Robinson, Saman Mukhtar, Shane D'Souza, Tina Dutt and Tracy Stocks. Royal Papworth Hospital NHS Foundation Trust: Joshua Wade, Lenka Cagova, Maksym Kovzel and Rachel Jooste. Sheffield Teaching Hospitals NHS Foundation Trust: Alison Delaney and Claire Mapplebeck. South Tees NHS Foundation Trust: Alycon Walker, Andrea Watson, Andrew Vaux, Asia Sawar, Carol Hannaway, Charlotte Jacobs, Claire Elliot, Claire Elliott, Craig Mower, Daiana Ferro, Emanuela Mahmoud, Gill Laidlaw, Julie Potts, Keith Harland, Laura Munglani, Lauren Fall, Leanne Murray, Lesley Harris, Lisa Wayman, Lisa Westwood, Louisa Watson, Lynne Naylor, Matthew Siddaway, Paula Robson, Rita Mohan, Sarah Essex, Sara Griffiths and Steven Liggett. University Hospital Southampton NHS Foundation Trust: Andreia Valente, Rashid Kazmi, Ruth Kirby, Sarah Bowmer and Yanli Li. University Hospitals Birmingham NHS Foundation Trust: Alice Longe, Amy Bamford, Anand Lokare, Andrew McDarby, Aneta Drozd, Cathy Stretton, Catia Mulvihill, Charlotte Ferris, Christopher McGhee, Claire McNeill, Colin Bergin. Daniella Lynch, Fionnuala Lenehan, Gerry Gilleran, Gillian Lowe, Graham McIlroy, Helen Jenner, Helen Shackleford, Isma Younis, Jaspret Gill, Jimmy Musngi, Joanne Dasgin, Joanne Gresty, Joseph Nyaboko, Juneka Begum, Katerine Festejo, Katherine Lucas, Katie Price, Khushpreet Bhandal, Kristina Gallagher, Kyriaki Tsakiridou, Lauren Cooper, Louise Wood, Lulu Amutike, Marie Thomas, Marwan Kwok, Melanie Kelly, Michelle Bates, Nafeesah Ahmad Haider, Nicholas Adams, Oliver Topping, Rachel Smith, Rani Maria Joseph, Salma Kadiri, Samantha Caddick, Samuel Harrison, Shereef Elmoamly, Stavroula Chante, Sumaiyyah Gauhar, Syed Ashraf, Tabinda Kharodia and Zhane Peterkin. University Hospitals of Leicester NHS Trust: Isgro Graziella and Hakeem Yusuff. University Hospitals of North Midlands NHS Trust: David Sutton, Ian Massey, Jade Di‐Silvestro, Joanne Hiden, Mia Johnson and Richard Buka. University Hospitals Plymouth NHS Trust: Claire Lentaigne, Jackie Wooding and Nicola Crosbie; Whittington Health NHS Trust: Ana Alvaro, Emma Drasar, Elen Roblin, Georgina Santiapillai, Kathryn Simpson, Kayleigh Gilbert, Yanrong Jiang, Zara Sayar and Zehraa Al‐Khafaji.

## References

[1] Hippensteel JA , LaRiviere WB , Colbert JF , Langouët‐Astrié CJ , Schmidt EP . Heparin as a therapy for COVID‐19: current evidence and future possibilities. Am J Physiol Lung Cell Mol Physiol. 2020;319(2):L211–l7. 32519894 10.1152/ajplung.00199.2020 PMC7381711

[2] Shen L , Qiu L , Liu D , Wang L , Huang H , Ge H , et al. The association of low molecular weight heparin use and in‐hospital mortality among patients hospitalized with COVID‐19. Cardiovasc Drugs Ther. 2022;36(1):113–20. 33394360 10.1007/s10557-020-07133-3 PMC7779648

[3] Goligher EC , Bradbury CA , McVerry BJ , Lawler PR , Berger JS , Gong MN , et al. Therapeutic anticoagulation with heparin in critically ill patients with Covid‐19. N Engl J Med. 2021;385(9):777–89. 34351722 10.1056/NEJMoa2103417 PMC8362592

[4] Lawler PR , Goligher EC , Berger JS , Neal MD , McVerry BJ , Nicolau JC , et al. Therapeutic anticoagulation with heparin in noncritically ill patients with Covid‐19. N Engl J Med. 2021;385(9):790–802. 34351721 10.1056/NEJMoa2105911 PMC8362594

[5] Boonyawat K , Chantrathammachart P , Numthavaj P , Nanthatanti N , Phusanti S , Phuphuakrat A , et al. Incidence of thromboembolism in patients with COVID‐19: a systematic review and meta‐analysis. Thromb J. 2020;18(1):34. 33292258 10.1186/s12959-020-00248-5 PMC7680990

[6] Malas MB , Naazie IN , Elsayed N , Mathlouthi A , Marmor R , Clary B . Thromboembolism risk of COVID‐19 is high and associated with a higher risk of mortality: a systematic review and meta‐analysis. EClinicalMedicine. 2020;29:100639. 33251499 10.1016/j.eclinm.2020.100639 PMC7679115

[7] Spyropoulos AC , Anderson FA Jr , FitzGerald G , Decousus H , Pini M , Chong BH , et al. Predictive and associative models to identify hospitalized medical patients at risk for VTE. Chest. 2011;140(3):706–14. 21436241 10.1378/chest.10-1944

[8] Amin AN , Varker H , Princic N , Lin J , Thompson S , Johnston S . Duration of venous thromboembolism risk across a continuum in medically ill hospitalized patients. J Hosp Med. 2012;7(3):231–8. 22190427 10.1002/jhm.1002

[9] von Meijenfeldt FA , Havervall S , Adelmeijer J , Lundström A , Rudberg AS , Magnusson M , et al. Prothrombotic changes in patients with COVID‐19 are associated with disease severity and mortality. Res Pract Thromb Haemost. 2021;5(1):132–41. 33537537 10.1002/rth2.12462 PMC7845083

[10] https://www.nice.org.uk/guidance/ng89/chapter/Recommendations. 2018.

[11] Kyriakoulis KG , Kollias A , Kyriakoulis IG , Kyprianou IA , Papachrysostomou C , Makaronis P , et al. Thromboprophylaxis in patients with COVID‐19: systematic review of national and International clinical guidance reports. Curr Vasc Pharmacol. 2022;20(1):96–110. 34431465 10.2174/1570161119666210824160332

[12] Schulman S , Sholzberg M , Spyropoulos AC , Zarychanski R , Resnick HE , Bradbury CA , et al. ISTH guidelines for antithrombotic treatment in COVID‐19. J Thromb Haemost. 2022;20(10):2214–25. 35906716 10.1111/jth.15808 PMC9349907

[13] Roberts LN , Whyte MB , Georgiou L , Giron G , Czuprynska J , Rea C , et al. Postdischarge venous thromboembolism following hospital admission with COVID‐19. Blood. 2020;136(11):1347–50. 32746455 10.1182/blood.2020008086 PMC7483432

[14] Schulman S , Kearon C . Definition of major bleeding in clinical investigations of antihemostatic medicinal products in non‐surgical patients. J Thromb Haemost. 2005;3(4):692–4. 15842354 10.1111/j.1538-7836.2005.01204.x

[15] Hunt BJ . Preventing hospital associated venous thromboembolism. BMJ. 2019;365:l4239. 31227478 10.1136/bmj.l4239 PMC6591776

[16] Page EM , Ariëns RAS . Mechanisms of thrombosis and cardiovascular complications in COVID‐19. Thromb Res. 2021;200:1–8. 33493983 10.1016/j.thromres.2021.01.005 PMC7813504

[17] Levi M , Thachil J , Iba T , Levy JH . Coagulation abnormalities and thrombosis in patients with COVID‐19. Lancet Haematol. 2020;7(6):e438–e40. 32407672 10.1016/S2352-3026(20)30145-9 PMC7213964

[18] Spyropoulos AC , Ageno W , Cohen AT , Gibson CM , Goldhaber SZ , Raskob G . Prevention of venous thromboembolism in hospitalized medically ill patients: a U.S. Perspective Thromb Haemost. 2020;120(6):924–36. 32492724 10.1055/s-0040-1710326

[19] Zayed Y , Kheiri B , Barbarawi M , Banifadel M , Abdalla A , Chahine A , et al. Extended duration of thromboprophylaxis for medically ill patients: a systematic review and meta‐analysis of randomised controlled trials. Intern Med J. 2020;50(2):192–9. 31276276 10.1111/imj.14417

[20] Giannis D , Allen SL , Tsang J , Flint S , Pinhasov T , Williams S , et al. Postdischarge thromboembolic outcomes and mortality of hospitalized patients with COVID‐19: the CORE‐19 registry. Blood. 2021;137(20):2838–47. 33824972 10.1182/blood.2020010529 PMC8032474

[21] Zuin M , Engelen MM , Barco S , Spyropoulos AC , Vanassche T , Hunt BJ , et al. Incidence of venous thromboembolic events in COVID‐19 patients after hospital discharge: a systematic review and meta‐analysis. Thromb Res. 2022;209:94–8. 34896917 10.1016/j.thromres.2021.11.029 PMC8648604

[22] Ramacciotti E , Barile Agati L , Calderaro D , Aguiar VCR , Spyropoulos AC , de Oliveira CCC , et al. Rivaroxaban versus no anticoagulation for post‐discharge thromboprophylaxis after hospitalisation for COVID‐19 (MICHELLE): an open‐label, multicentre, randomised, controlled trial. Lancet. 2022;399(10319):50–9. 34921756 10.1016/S0140-6736(21)02392-8 PMC8673881

[23] Wang TY , Wahed AS , Morris A , Kreuziger LB , Quigley JG , Lamas GA , et al. Effect of Thromboprophylaxis on clinical outcomes after COVID‐19 hospitalization. Ann Intern Med. 2023;176(4):515–23. 36940444 10.7326/M22-3350 PMC10064277

[24] Kaatz S , Ahmad D , Spyropoulos AC , Schulman S . Definition of clinically relevant non‐major bleeding in studies of anticoagulants in atrial fibrillation and venous thromboembolic disease in non‐surgical patients: communication from the SSC of the ISTH. J Thromb Haemost. 2015;13(11):2119–26. 26764429 10.1111/jth.13140

