# Supplementary material for: Impact of thromboprophylaxis on hospital acquired thrombosis following discharge in patients admitted with COVID‐19: Multicentre observational study in the UK
Source: Br J Haematol. 2023 May 18;202(3):485–97. doi: 10.1111/bjh.18874 (PMC10952807; doi:10.1111/bjh.18874)
Supplement: Supplementary file 1 — Data S1. [file BJH-202-485-s001.docx]

**Supplementary material**

**Data collected using pre-designed electronic CRF.**

Patient demographics, comorbidities, laboratory parameters at the admission, anticoagulant treatment (prophylactic dose or treatment dose LWMH or unfractionated heparin), treatment with steroids, Blood transfusion during admission, clinical outcomes (all-cause mortality, thrombosis, major bleeding, clinically relevant non-major bleeding, Heparin induced thrombocytopenia, multiorgan failure, renal failure, secondary infection) admission to intensive care units, requirement for mechanical ventilation were collected during the hospital admission.

**Data management**

Multiple imputation was used to account for missing laboratory values (<10%) but not for comorbidities or clinical outcomes. The multiple imputation by chained equation (MICE) technique with its regression imputation model was used for this imputation with ten iterative cycles. Once imputation was done, results were reviewed for each imputed feature to make sure that the imputation has generated plausible data. (Scatterplots for the imputed features were used for the review.) We used variables which are predictive of missing values by considering a MAR (Missing At Random) assumption, which means that the probability that a value is missing depends only on observed values and not on unobserved values. All tests were two sided, and P values <0.05 were deemed statistically significant. All analyses were performed using either SPSS version 27 (SPSS v27; IBM, Armonk, NY, USA), R (v4.0.3, Open-source software) or Stata (v17, StataCorp LLC, College Station, TX, USA) and open-source software programming languages and libraries (Python [v3.7, Open-source software], panda [v1.3.3, Open-source software], numpy [v1.21.2, Open-source software], scikit learn [v0.22.1, Open-source software]).
